# Evolution of Renewable Energy in BRI Countries: A Combined Econometric and Decomposition Approach

**DOI:** 10.3390/ijerph17228668

**Published:** 2020-11-22

**Authors:** Feng Dong, Yuling Pan

**Affiliations:** School of Economics and Management, China University of Mining and Technology, Xuzhou 221116, China; ts19070137a31ld@cumt.edu.cn

**Keywords:** renewable energy, Belt and Road Initiative countries, *β*-convergence, Kaya equation, LMDI

## Abstract

The development of renewable energy is an important cooperation theme among countries along the Belt and Road Initiative (BRI countries). Through map description, we first explore the changes in renewable energy consumption in BRI countries. Then, *β*-convergence is employed to examine the development direction of renewable energy consumption in BRI countries. Finally, based on the expanded Kaya equation, we decompose the factors effecting renewable energy consumption into energy structure effect, energy intensity effect, low-carbon economic effect, carbon emission effect, population distribution effect and population effect. The Logarithmic Mean Divisia Index (LMDI) is utilized to calculate the contribution of each factor to renewable energy consumption in the expanded Kaya equation. Our research reaches the following conclusions: (1) *β*-convergence exists in renewable energy consumption among BRI countries, indicating that it will converge to a relatively stable level, and countries with low renewable energy consumption will increase their renewable energy consumption with a higher convergence rate to chase the countries with high renewable energy consumption. (2) Energy structure effect makes a positive impact on renewable energy consumption, and is the main contributor to renewable energy consumption. (3) The energy intensity effect makes a negative contribution to renewable energy consumption, and the negative impact has deepened in recent years. (4) Both the low-carbon economic effect and the carbon emission effect have positive impacts on renewable energy consumption. Our research not only provides a description of the experience of developing renewable energy for BRI countries, but also makes reference to other organizations.

## 1. Introduction

Renewable energy is a critical part of contemporary energy consumption and an important field for future energy development. Adequate, safe and sustainable energy is the basic guarantee of national economic development and social progress [1]. At present, many countries are faced with the problems of low energy consumption per capita and high pressure on energy demand, which have led to conflicts between energy supply and economic development, especially in developing countries [2]. The development of renewable energy can save resources, ease energy pressure and realize the sustainable development of energy. Moreover, renewable energy development can also protect the environment and reduce pollution emissions, which is an important measure to deal with the future climate change. Since industrialization, the proportion of primary energy (e.g., oil, coal, etc.) in energy consumption has been high and various pollutant gases (e.g., SO_2_, NO_2_, etc.) have been emitted during the energy use process. In particular, the greenhouse gas CO_2_ is mainly derived from the use of primary energy [3,4]. The development of renewable energy plays a very important role in optimizing energy structure, protecting the environment and responding to climate change. In addition, renewable energy development can expand new economic areas [5], increase employment options [6] and promote green economic transformation [7]. 

Renewable energy development relies on natural resources and geographical characteristics. For example, tidal energy depends on marine characteristics, and solar energy depends on climate characteristics. This suggests that renewable energy development requires local resources, which is of great significance when attempting to expand employment and promote economic development.

The Belt and Road Initiative adheres to the principles of sharing and joint construction, which are devoted to creating a new economic “normal” and establishing business on the basis of access and navigation [8]. The BRI countries are rich in renewable energy resources and have huge potential for renewable energy development [9]. For example, Kazakhstan has a wind power generation potential of 182 billion kW/year, Kyrgyzstan has a solar power generation potential of 23.4 kW·h/m^2^, and Turkmenistan is rich in solar resources because the country has an average of 300 days of sunshine per year. In addition, renewable energy development also has the support of policy systems in BRI countries, including legal documents, such as the “Support for the Use of Renewable Energy and Electricity Law” (Kazakhstan), departmental regulatory documents, such as the “Rules for the Use of Renewable Resources on the Equipment List” (Tajikistan), and documents related to national development strategies, such as “Conversion to a Green Economy Concept” (Kazakhstan), the “Medium-Term Electricity Development Strategy” (Kyrgyzstan), etc. The Belt and Road Initiative provides a space for cooperation in renewable energy development and investment [10]. 

**Figure 1:**
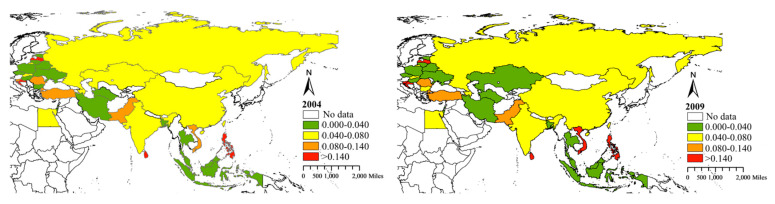

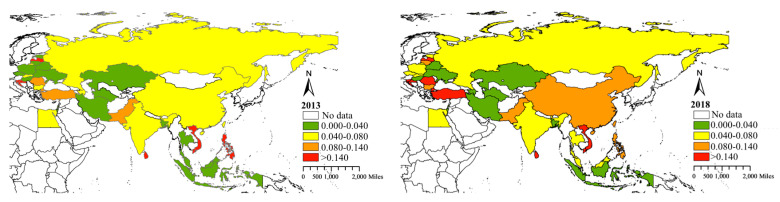
Share of renewable energy consumption in Belt and Road Initiative (BRI) countries.

Energy security, clean production and environmental protection are the foundations of energy and environmental development. In recent years, the proportion of renewable energy consumption within the total energy consumption of BRI countries has changed significantly. As can be seen in Figure 1, if 0.04, 0.08 and 0.14 are taken as the demarcation values for the low, lower, higher and high categories for the share of renewable energy consumption respectively, then the results show that the share of renewable energy consumption are low in 2004, 2009 and 2013 in most countries, except Vietnam (abundant hydropower and wind energy), Philippines (tidal energy), Sri Lanka (hydropower), Pakistan (hydropower), Turkey (hydropower), Romania (hydropower), Lithuania (hydropower), Austria (hydropower), and Croatia (hydropower). From 2013–2018, the proportion of renewable energy consumption in China, Thailand, Turkey and other countries has increased significantly, which is closely related to these countries’ development policies. For example, India has implemented a bidding mechanism for renewable energy power generation and has reduced tariffs on associated raw materials. Vietnam has introduced tax exemptions for the import of fixed assets associated with solar energy projects and has reduced the relevant fees for land and water use. Some renewable energy projects also enjoy financing concessions from the World Bank, the Asian Development Bank and the European Bank for Reconstruction and Development.

As of 2018, the average share of total energy consumption represented by renewable energy in BRI countries is 9.48%. Among them, the country with the highest renewable energy consumption share is Croatia, where it accounts for 25.27% of total energy consumption. However, in terms of economic development and environmental protection, the International Renewable Energy Agency states that by 2050 the proportion of global renewable energy consumption must exceed 2/3 [11]. This suggests that the development of renewable energy in BRI countries still has a long way to go. Small investment scales, low cooperation level and intense electricity price competition are the main issues affecting the development of renewable energy in BRI countries, especially in Central Asia and Africa. The current development of renewable energy in BRI countries is still at the initial stage.

The above introduction shows that the BRI countries have huge potential for renewable energy development. Meanwhile, BRI countries have strongly supported renewable energy development, such as proposing renewable energy development policies and introducing renewable energy projects. However, from the perspectives of energy conservation and environmental protection, the development of renewable energy in BRI countries still requires long-term efforts. 

**Figure 2:**
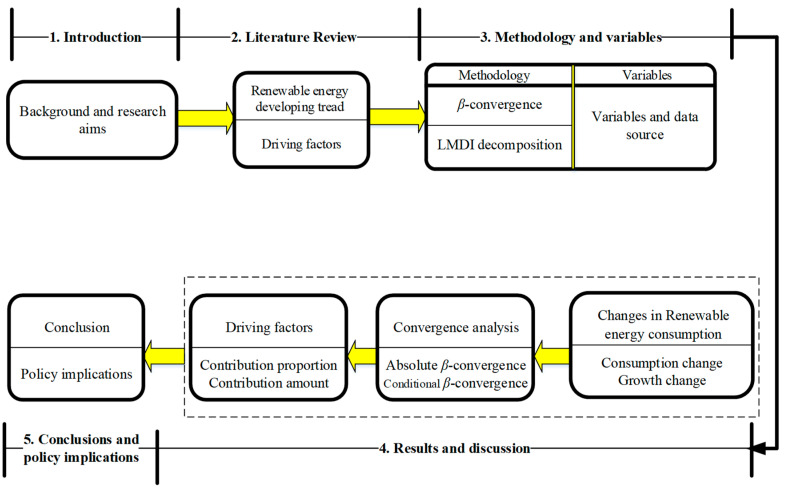
Framework of this study.

We are interested in two issues. First, what are the developing trends and characteristics of renewable energy consumption in BRI countries? Second, what factors drive the development of renewable energy consumption in BRI countries? The specific idea is shown in Figure 2.

To address the above issues, this paper is structured as follows. Firstly, *β*-convergence proves that many developing countries are catching up with the economy of developed countries at a faster rate. This paper utilizes *β*-convergence analysis to explore whether renewable energy consumption in BRI countries shows such a development trend. Then, the paper utilizes a decomposition model to attribute changes in renewable energy consumption to changes in multiple factors, and calculates the contribution of each factor, thereby discussing the driving factors of renewable energy consumption. The purpose of this study is to explore the development trends and driving factors of renewable energy in BRI countries, thereby assisting renewable energy development in other countries or alliance organizations.

## 2. Literature Review

Researchers across the globe have been studying renewable energy since the 20th century, especially its development potential. Many researchers believe that renewable energy is an important strategy for future environmental management [12,13]. In contrast, some researchers have analyzed the regional development prospects for renewable energy and regarded renewable energy development as an important strategy for solving energy consumption problems [14,15]. Others believe that the future development of renewable energy will greatly affect regional economic development [16]. With the continuous development of the industrial economy, renewable energy is considered to be one of the main solutions to the current environmental and energy consumption problems [17,18,19]. The development of renewable energy has led to many related studies such as the consumption of renewable energy and its influencing factors.

### 2.1. Development Trends in Renewable Energy

Previous studies have shown that the development of renewable energy in developed countries is much faster than in developing countries, and different development stages have led to different development trends [20]. Policy is considered to be an invisible hand guiding energy consumption [21]. Murshed [22] analyzes low, lower-middle and upper-middle income countries across the five continents. The results show that the development of renewable energy mainly depends on their renewable trade liberalization policies, and the renewable energy transition period for upper-middle income countries is shorter than that in low and lower-middle income countries. Similarly, Chen et al. [23] suggest that renewable energy development in developing countries is closely related to renewable energy investment, and argue that only when renewable energy investment reaches a certain threshold can renewable energy development be significantly improved. However, most developing countries are limited by trade liberalization policies, and there are feedback links and interdependence between income, renewable energy development and trade. Besides, Amri [24] suggests that fiscal incentives should be applied to promote renewable energy project investment. In addition to the national development stage, Carfora et al. [25] suggest that renewable energy development is closely related to national development policies. In 1993, Japan implemented the “New Sunshine Plan”, which will inform renewable energy development for at least the next 70 years [26]. In 1997, the European Union issued a white paper on renewable energy development and formulated the European Union strategy for renewable energy development up to 2050 [27]. In 2006, A European Strategy for Sustainable, Competitive Secure Energy addressed the fact that Europe has entered the era of new energy [28]. In 2011, the European Commission released the Energy Roadmap 2050, once again proposing an energy strategy path towards the year 2050 [29]. As a result, the European Union is the fastest growing region for renewable energy in the world [30]. The United States has aligned energy issues with national development strategies and conducted research into renewable energy and other clean energy fields [31]. China included a renewable energy development strategy in its 2004 comprehensive energy development strategy, which planned to fully realize the commercialization of renewable energy by 2050. Compared to 2004, renewable energy consumption in Japan, the European Union, the United States and China increased by 113.26%, 95.14%, 83.42% and 149.01%, respectively, while global renewable energy consumption increased by 61.94% in 2018. Therefore, improving the policy systems related to renewable energy can increase renewable energy development levels [32].

The concept of convergence is proposed by Baumol [33] and is used to analyze convergence of the productivities shown by industrialized market economies. Convergence analysis includes *σ*-convergence, *β*-convergence and club convergence. *β*-convergence assumes that the growth rates in underdeveloped regions are greater than in developed regions, and the development level of these regions will reach a relatively stable level in the future [34]. This characteristic is frequently used in research of the energy field. Hao and Peng [35] study the convergence of per capita energy consumption in China and show that there is absolute *β*-convergence and conditional *β*-convergence for per capita energy consumption in 30 provinces across China. The energy density of the BRI countries has also been shown to be convergent, and the increase in globalization has accelerated energy density convergence in BRI countries [36]. *β*-convergence is widely used in the field of energy research to explore the convergence trend for energy in different regions during energy development, in other words the energy development balance between regions. When *β*-convergence is proved, it indicates that energy development within each region in an area will probably be consistent in the future, and overall energy development will tend to be balanced [37].

### 2.2. Drivers of Renewable Energy Consumption

The Kaya equation was proposed by Kaya [38] at the first Intergovernmental Panel on Climate Change seminar and it was initially used to analyze the decomposition of CO_2_ emissions. Later, decomposition based on the Kaya equation was applied to other fields, such as energy consumption decomposition [39] and haze emission decomposition [40], etc. Therefore, the Kaya equation can also be introduced to the decomposition of renewable energy consumption. The driving factor most directly related to renewable energy consumption is energy structure, because controlling the consumption of fossil fuels is a direct driving force for the development of renewable energy [41]. In addition to energy structure, energy intensity also affects the development of renewable energy [42]. The higher the energy intensity, the greater the demand for energy, and due to limited resources the greater the demand for renewable energy. Furthermore, carbon emission also has an impact on renewable energy consumption. With more than 150 countries signing the Kyoto Protocol, global carbon emissions continue to decrease. Primary energy consumption is the main source of carbon emissions, therefore renewable energy development is the best choice when attempting to mitigate climate change without affecting the economy [43]. Furthermore, reducing carbon emissions and developing a low-carbon economy can promote the development of renewable energy [44]. The demographic factor is also an important factor affecting renewable energy consumption. About one-seventh of the world population has no access to electricity, and solving the electricity problem is an important challenge for current energy consumption strategies. In the context of current energy consumption and environmental protection, the problem of electricity demand in the residential sector needs to be addressed by renewable energy development [45].

The combination of the expanded Kaya equation and LMDI decomposition is widely used in various research to calculate the contribution of a variable using different driving factors [46]. To deal with the continuous multiplication form of the expanded Kaya equation, LMDI can be decomposed by addition or multiplication. Its advantages are no residual term and reversibility. Wang et al. [47] apply LMDI decomposition to analyze the factors affecting energy consumption in Hunan Province (China) and divide the influencing factors into scale effect, structural effect, and efficiency effect. The results show that the scale effect promotes the rapid growth of energy consumption. Coal consumption growth in China is broken down into population, economic effects, energy intensity and energy structure effects. The results show that the economic effect is the main force driving coal consumption growth [48]. In addition to the regional level, LMDI decomposition can also be used to decompose energy consumption at the industry level. Wang and Feng [49] discuss the main factors affecting energy consumption changes in the non-ferrous metal industry. They decompose energy consumption into energy structure effect, energy intensity effect, industrial structure effect, labor production effect and industrial scale effect. The results show that the labor production effect is the main contributor to energy consumption changes. The energy consumption changes in the Chinese construction industry are broken down into key factors, the operating factor and the geographical factor, and the results show that the operating factor is the main factor affecting energy consumption changes in the construction industry [50].

Through a review of the existing renewable energy development trends and related literature on the factors driving energy consumption, the unique contributions of this study are as follows. (1) This study analyzes the developing trend of renewable energy consumption in BRI countries. (2) The expanded Kaya equation is utilized to decompose the changes in renewable energy consumption, and summarize the influencing factors affecting renewable energy consumption into energy structure effect, energy intensity effect, low-carbon economic effect, carbon emission effect, population distribution effect and population effect. Furthermore, the LMDI is applied to solve the contributions made by various factors to renewable energy consumption changes in BRI countries. This review mainly explores the developing trend and driving factors of renewable energy consumption in BRI countries, and provides experience that can be used to improve the development of renewable energy in other regions and organizations.

## 3. Methodology

### 3.1. Convergence Analysis Based on Dynamic Panel Data

Before exploring the driving factors of renewable energy consumption, there are some problems that need to be addressed. What is the developing trend of renewable energy consumption in BRI countries, and is there any difference between before and after the implementation of the Belt and Road Initiative? In response to the above problems, we use the convergence analysis method to explore the renewable energy development trends in BRI countries.

Based on Baumol’s research [33], this review designs a convergence model that can be used to study the development of renewable energy in BRI countries, as shown in Formula (1).

(1) $$\ln(RE_{i,t})=\varphi \ln(RE_{i,t-1})+\gamma x_{i,t}+\delta +\mu_{i,t}$$

where *RE_i_*_,*t*_ represents the renewable energy consumption of the *i_th_* country in year *t*, δ represents a constant term, *μ_i_*_,*t*_ represents an error term, and *x_i_*_,*t*_ represents a vector composed of control variables, and is coefficient of *x_i_*_,*t*_. 

The sample in this review is a dynamic panel. In order to eliminate the non-observed effects, this review differentiates the horizontal equation and further obtains the difference equation, as shown in Formula (2).

(2) $$\Delta \ln(RE_{i,t})=\varphi \Delta \ln(RE_{i,t-1})+\Delta \gamma x_{i,t}+\Delta \mu_{i,t}$$

Among them, φ represents the coefficient of β-convergence, and has the following meanings:

When φ < 1, the development of renewable energy consumption shows a convergent trend;

When φ > 1, renewable energy consumption presents a divergent trend;

When φ = 1, the renewable energy consumption is in equilibrium.

In addition, if there is a convergence in the renewable energy consumption of the sample countries, then the convergence rate *v* can be calculated by *v* = −*ln(φ)*.

The dynamic panel has characteristics of short timings, with large cross sections after the overall time period is divided, so it is unreasonable to apply the Fixed Effect to solve Formulas (1) and (2), because the estimated results of the Fixed Effect show a large deviation. Therefore, the GMM (generalized method of moments) estimator is introduced to deal with the formulas, which also considers the problem of variable endogeneity [51]. 

In Formulas (1) and (2), the vector *x_i_*_,*t*_ includes *PGDP, Ur, FDI* and *Ind*. The specific explanations of each variable are shown in Table 1. In recent years, more and more countries have joined the Belt and Road Initiative [52]. The sample for this study is from 2004 to 2018, but many countries do not publish renewable energy consumption data in the early years. Therefore, only 32 BRI countries are selected as the samples in this review, including Bulgaria, Croatia, Czech Republic, Estonia, Hungary, Latvia, Lithuania, Poland, Romania, Slovakia, Slovenia, Turkey, Ukraine, Azerbaijan, Belarus, Kazakhstan, Russian Federation, Turkmenistan, Iran, Israel, Egypt, Bangladesh, China, India, Indonesia, Malaysia, Pakistan, Philippines, Singapore, Sri Lanka, Thailand and Vietnam.

### 3.2. LMDI Decomposition

Aiming at the driving factors of renewable energy consumption in BRI countries, we are interested in two problems. First, what are the driving factors affecting renewable energy consumption? Second, what are the differences in these driving factors in recent years? This paper adapts the expanded Kaya equation to decompose the driving factors of renewable energy consumption. Since there has been no previous research on the decomposition of renewable energy based on the Kaya equation, drawing on other decomposition studies in the energy field [57], the decomposition equation for renewable energy consumption is shown in Formula (3) and the variable explanations are shown in Table 2.

(3) $$\begin{array}{ll} RE=\sum_{i}RE_{i} & =\sum_{i}\frac{RE_{i}}{E_{i}}\times \frac{E_{i}}{G_{i}}\times \frac{G_{i}}{C_{i}}\times \frac{C_{i}}{P_{i}}\times \frac{P_{i}}{P}\times P\\ & =\sum_{i}RS_{i}\times EI_{i}\times CE_{i}\times PC_{i}\times PP_{i}\times P \end{array}$$

where $RS_{i}=\frac{RE_{i}}{E_{i}}$ represents the energy structure. The larger the *RS_i_*, the more a country attaches importance to the development of renewable energy, such as wind energy, solar energy and biomass energy, etc. $EI_{i}=\frac{E_{i}}{G_{i}}$ represents energy intensity. The larger the *EI_i_*, the higher the dependence of a country on energy consumption during the economic development process. $CE_{i}=\frac{G_{i}}{C_{i}}$ represents the low-carbon economy development level. When GDP is the same, if *CE_i_* is large, then carbon emissions are low, which indicates that a country has a higher low-carbon economy development level. $PC_{i}=\frac{C_{i}}{P_{i}}$ represents carbon emissions per capita. The larger the *PC_i_*, the greater the demand for carbon emissions per capita. $PP_{i}=\frac{P_{i}}{P}$ represents the proportion due to population and indicates the population distribution of BRI countries.

In the expanded Kaya equation, the factors affecting the changes in renewable energy consumption are decomposed into the energy structure effect, energy intensity effect, low-carbon economic effect, carbon emission effect, population distribution effect and population effect. These factors not only have an impact on renewable energy consumption in mathematical models, but also have an impact in an economic sense. In recent years, countries have made considerable attempts to improve primary energy conservation and green energy consumption. Adjusting the energy structure and reducing the energy intensity are the most direct methods of achieving these goals [58]. Zhu et al. [59] suggest that adjustments to the energy structure require the development of energy diversity, especially renewable energy diversity. Reducing energy intensity and improving energy efficiency can promote the development of energy diversity. In addition, the development of renewable energy is conducive to reducing carbon emissions. As an important globalization theme, the development of a low-carbon economy also plays a vital role in the development of renewable energy [60]. A decrease in per capita carbon emissions will improve renewable energy consumption in a similar way to the development of a low-carbon economy. Population is an important factor that affects such aspects as CO_2_ emissions, energy consumption and economic growth [61]. Therefore, it is reasonable to introduce the effects of population distribution and the population effect into the models.

The LMDI is the most popular time series data method in the Index Decomposition Analysis method. It can solve the zero value in the decomposition variables and eliminate the influence of residuals on the decomposition results. In order to solve Formula (10), we utilize the LMDI to solve the contributions made by various factors to renewable energy consumption. It includes multiplicative decomposition and additive decomposition, which can convert into each other. We use additive decomposition to analyze the effects of energy structure, energy intensity, low-carbon economy, carbon emissions, population distribution and population effect on renewable energy consumption in BRI countries between 2004–2018. Changes in renewable energy consumption between the base period (0) and year *T* are shown by:

(4) $$\Delta RE=RE^{T}-RE^{0}$$

According to the LMDI, Formula (4) can be calculated by:

(5) $$\Delta RE=\Delta RE_{RS}+\Delta RE_{EI}+\Delta RE_{CE}+\Delta RE_{PC}+\Delta RE_{PP}+\Delta RE_{P}$$

where $\Delta RE_{RS}$ indicates the changes in renewable energy consumption due to energy structure; $\Delta RE_{EI}$ indicates the changes in renewable energy consumption due to energy intensity; $\Delta RE_{CE}$ indicates the changes in renewable energy consumption due to the low-carbon economy development level; $\Delta RE_{PC}$ indicates the changes in renewable energy consumption due to carbon emissions per capita; $\Delta RE_{PP}$ indicates the changes in renewable energy consumption due to population distribution; and $\Delta RE_{P}$ indicates the changes in renewable energy consumption due to population size. Each of these can be calculated by Formulas (6)–(11).

(6) $$\Delta RE_{RS}=\left\{ \begin{array}{cc} \sum_{i}L(RE_{i}^{T},RE_{i}^{0})\times \ln\left(\frac{RS_{i}^{T}}{RS_{i}^{0}}\right) & ,RS_{i}^{T}\times RS_{i}^{0}\neq 0\\ 0 & ,RS_{i}^{T}\times RS_{i}^{0}=0 \end{array}\right.$$

(7) $$\Delta RE_{EI}=\left\{ \begin{array}{cc} \sum_{i}L(RE_{i}^{T},RE_{i}^{0})\times \ln\left(\frac{EI_{i}^{T}}{EI_{i}^{0}}\right) & ,EI_{i}^{T}\times EI_{i}^{0}\neq 0\\ 0 & ,EI_{i}^{T}\times EI_{i}^{0}=0 \end{array}\right.$$

(8) $$\Delta RE_{CE}=\left\{ \begin{array}{cc} \sum_{i}L(RE_{i}^{T},RE_{i}^{0})\times \ln\left(\frac{CE_{i}^{T}}{CE_{i}^{0}}\right) & ,CE_{i}^{T}\times CE_{i}^{0}\neq 0\\ 0 & ,CE_{i}^{T}\times CE_{i}^{0}=0 \end{array}\right.$$

(9) $$\Delta RE_{PC}=\left\{ \begin{array}{cc} \sum_{i}L(RE_{i}^{T},RE_{i}^{0})\times \ln\left(\frac{PC_{i}^{T}}{PC_{i}^{0}}\right) & ,PC_{i}^{T}\times PC_{i}^{0}\neq 0\\ 0 & ,PC_{i}^{T}\times PC_{i}^{0}=0 \end{array}\right.$$

(10) $$\Delta RE_{PP}=\left\{ \begin{array}{cc} \sum_{i}L(RE_{i}^{T},RE_{i}^{0})\times \ln\left(\frac{PP_{i}^{T}}{PP_{i}^{0}}\right) & ,PP_{i}^{T}\times PP_{i}^{0}\neq 0\\ 0 & ,PP_{i}^{T}\times PP_{i}^{0}=0 \end{array}\right.$$

(11) $$\Delta RE_{P}=\left\{ \begin{array}{cc} \sum_{i}L(RE_{i}^{T},RE_{i}^{0})\times \ln\left(\frac{P_{}^{T}}{P_{}^{0}}\right) & ,P_{}^{T}\times P_{}^{0}\neq 0\\ 0 & ,P_{}^{T}\times P_{}^{0}=0 \end{array}\right.$$

Among them, $L(RE_{i}^{T},RE_{i}^{0})=\left\{ \begin{array}{cc} \frac{RE_{i}^{T}-RE_{i}^{0}}{\ln RE_{i}^{T}-\ln RE_{i}^{0}} & ,RE_{i}^{T}\neq RE_{i}^{0}\\ RE_{i}^{T} & ,RE_{i}^{T}=RE_{i}^{0} \end{array}\right.$.

## 4. Results and Discussion

### 4.1. Convergence Analysis

Before regression, this paper introduces the unit root test and cointegration test to the panel data. The results show that the horizontal series of some variables have unit roots, and the first-order difference series of all variables do not have unit roots, i.e., the panel data is stable. Further, the cointegration test shows that there is a significant cointegration relationship between variables. Table 3 shows *β*-convergence regression

Table 3 shows the β-convergence results for the dynamic panel data. Models (1)–(4) examine whether β-convergence exists during the period 2004–2018. The Wald chi2 test is used to determine the property degree of the model. In our models, all the tests of Wald chi2 are passed by rejecting the null hypothesis at the significance level of 0.01. The Arellano-Bond test is also performed because the GMM estimator assumes that residual terms have no autocorrelation with variables. Therein, AR(1) should reject the null hypothesis, while AR(2) should accept the null hypothesis. In addition, The Sargan test is performed to determine the correlations between variables and residual terms. In our models, both the Arellano-Bond test and Sargan test have been passed, indicating that it is reasonable to adapt the GMM estimator to solve the models.

The first row of Table 3 represents the coefficient φ of *lnRE_i,t_*_-1_. During the period 2004–2018, all the coefficients for *lnRE_i,t_*_-1_ are smaller than 1 at the 0.01 significance level, indicating that renewable energy consumption in BRI countries will converge to a relatively stable level during 2004–2013. However, the convergence rate varies depending on the different control variables.

Per capita GDP had a negative impact on the convergence rate for renewable energy consumption in BRI countries during 2013–2018, suggesting that the higher the per capita GDP, the slower the convergence rate for renewable energy consumption. Among BRI countries, most countries are developing countries. In the Kyoto Protocol, developing countries are not responsible for carbon reduction. The research and development costs and production costs of renewable energy are much higher than fossil energy. Therefore, developing countries will give priority to fossil energy when consuming energy. Ocal and Aslan [62] come to a similar conclusion. They suggest that the reason for this relationship is that renewable energy is expensive in developing countries, which means that the development trend for renewable energy will slow down as the needs of the economy increase, therefore economic development decreases the convergence rate for renewable energy consumption in developing countries.

The coefficient for urbanization is positive, indicating that urbanization accelerates the convergence rate for renewable energy consumption in BRI countries. For BRI countries, the implementation of the Belt and Road Initiative has not only brought about the development of infrastructure construction, but also the development of tourism (in recent years, the number of tourists coming to BRI countries has increased, especially Chinese tourists) [63], which puts forward high demands on the urban environment. Brito et al. [64] believe that the current direction of urban construction is towards a green and environmentally friendly city, which limits the discharge of industrial pollution and greatly increases the demand for green energy.

The coefficient for foreign direct investment is negative, suggesting that foreign direct investment slows down the convergence rate for renewable energy consumption in BRI countries. Amri [24] proposes that foreign direct investment improves renewable energy consumption in developed countries, while foreign direct investment can lead to the discovery of non-directional links in developing countries. 

The coefficient for industrialization is positive, suggesting that industrialization accelerates the convergence rate for renewable energy consumption in BRI countries. After the implementation of the Belt and Road Initiative, renewable energy cooperation has also greatly promoted the industrial development of BRI countries. As we can see, although developing countries do not bear the responsibility for carbon reduction, many developing countries have formulated their own emission reduction plans for the long-term development of the country, such as China [65], India [66], etc. Therefore, in the long run, industrial development will inevitably be decoupled from fossil energy and turn to renewable energy [67].

In view of *β*-convergence analysis, convergence exists in the renewable energy consumption of BRI countries, which means that the renewable energy consumption in BRI countries will converge to a relatively stable level. Furthermore, under the control variables, both urbanization and industrialization have positive impacts on the convergence rate for renewable energy consumption, while per capita GDP and FDI have negative impacts on renewable energy consumption.

### 4.2. Logarithmic Mean Divisia Index Decomposition Analysis

#### 4.2.1. Changes in the Factors Affecting Renewable Energy Consumption

**Figure 3:**
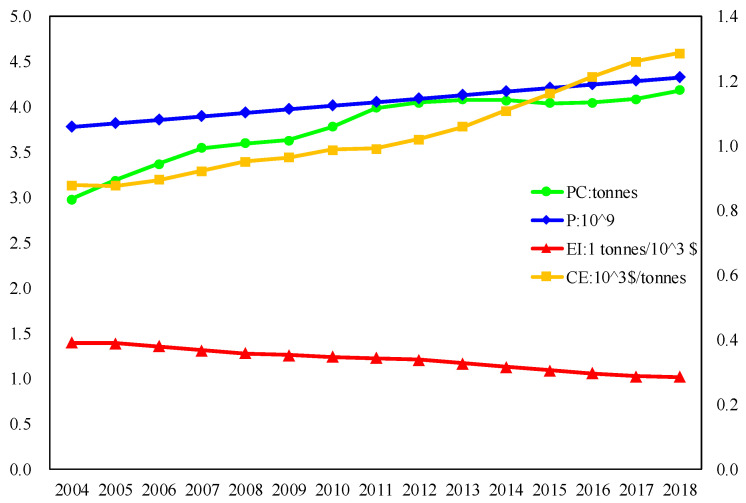
Changes in factors during 2004–2018.

Figure 3 lists the overall changes to the influencing factors in Formula (3) for BRI countries. Therein, the energy structure is listed in Figure 3. In addition, the population distribution is not suitable for overall analysis, so it is not shown in the figure. It can be seen that energy intensity is decreasing year by year, which indicates that the energy consumed per GDP is gradually decreasing in BRI countries. Especially after 2011, the trend for energy intensity reduction is more obvious. The low-carbon economy level is gradually rising in BRI countries, and the low-carbon economy improvement rate after 2011 is significantly higher than before. Carbon emissions per capita gradually increases in BRI countries, but after 2011 the growth rate for carbon emissions per capita significantly slows down. The population gradually increases in BRI countries over the time period used in this study, and this growth trend is almost linear.

#### 4.2.2. Decomposition Analysis

Table 4 shows the contribution rate for each factor.

**Figure 4:**
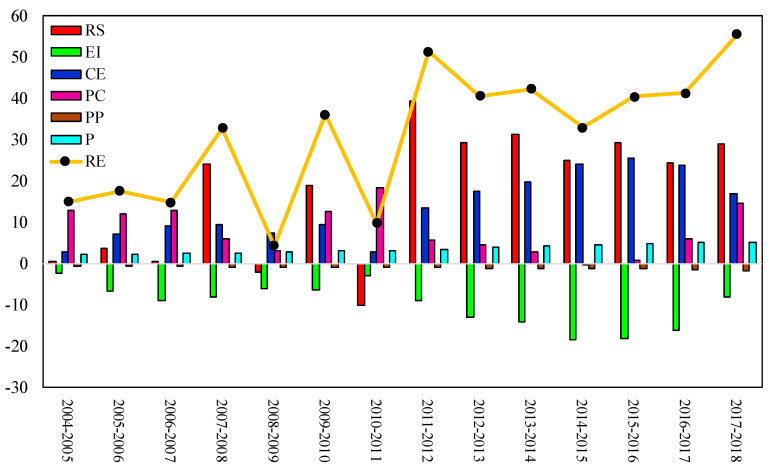
Specific contribution amount of each factor.

The LMDI decomposition is used to solve the Kaya equation and explore the temporal variation in renewable energy consumption of BRI countries between 2004–2018. Table 4 shows the contribution rate for each factor, and Figure 4 shows the specific contribution made by each factor. It can be seen that the change in renewable energy consumption has gradually increased year by year, especially after 2011 when the gap becomes significantly larger than before, indicating that implementation of the Belt and Road Initiative has effectively promoted the development of renewable energy. However, it is not clear what causes this rapid development.

Figure 4 also shows the contribution made by the six factors to the changes in renewable energy consumption. Therein, energy structure is the main factor affecting the variation in renewable energy consumption because it represents about 70% of the total. Between 2004–2018, the contribution rate for energy structure fluctuates to a certain extent, but the contribution amount gradually stabilized after 2012, indicating that the implementation of the Belt and Road Initiative has an adjustment effect on the energy structure of BRI countries. In recent years, regional energy cooperation has helped the BRI countries adjust their energy structure, which is mainly reflected in the increase of thermal power, wind power, hydropower, and solar power projects [68]. Adjusting the energy structure has a vital impact on renewable energy consumption, thereby further improving the renewable energy consumption in BRI countries.

Changes in energy intensity have a negative effect on the growth of renewable energy consumption, which means that reduction in energy intensity reduces the development of renewable energy. Especially after 2012, the negative contribution rate for energy intensity stabilizes by about 40%, and the negative contribution made by energy intensity significantly increases. In recent years, the energy intensity of the BRI countries has gradually decreased, which means that energy consumption demand per unit of GDP and renewable energy intensity also gradually decreases. Therefore, energy intensity now has a greater negative effect on the development of renewable energy.

The low-carbon economy has made a positive contribution to the development of renewable energy. Its contribution rate is about 30% before 2012 and about 60% after 2012. The contribution made by the low-carbon economy also significantly increases. Cheng et al. [69] believe that the development of a low-carbon economy will increase renewable energy consumption. Conversely, the consumption of renewable energy will also affect the development of the low-carbon economy, and there is a synergistic effect between them. 

The carbon emission effect has made a positive contribution to the development of renewable energy. Xu et al. [70] suggest that adjusting the energy structure can help developing countries reduce CO_2_ emissions and achieve a low-carbon economic transition. A low-carbon economic transition can drive the increase in renewable energy consumption. Compared with fossil energy, renewable energy is regarded as clean energy, because renewable energy hardly emits greenhouse gases, which is of great significance for developing a low-carbon economy and responding to global climate change. Low-carbon development is the vision of the Belt and Road Initiative, which can be summarized as “Improving the ecological environment, responding to climate change, and building a green silk road” [71]. As a result, the implementation of the Belt and Road Initiative has promoted the coordinated development of carbon emission reductions. However, after the implementation of the Belt and Road Initiative, the contribution rate made by the carbon emission effect has gradually decreased along with the contribution amount, which indicates that the implementation of the Belt and Road Initiative has reduced the impact of the carbon emission effect on renewable energy consumption.

The population distribution effect makes a negative contribution to renewable energy consumption, but the negative contribution rate decreased significantly after the implementation of the Belt and Road Initiative. However, it is clear, from the perspectives of contribution rate and contribution amount, that the impact of population distribution on renewable energy consumption is very small. In contrast, the population effect has always made a positive contribution to renewable energy consumption, and its contribution rate has not changed significantly, but the contribution amount has gradually increased. Especially after 2012, the positive contribution of the population effect to renewable energy consumption increases significantly, which suggests that the implementation of the Belt and Road Initiative has not increased the contribution rate of the population effect to renewable energy, but has increased the overall size of the corresponding contribution (The contribution rate indicates the proportion of the contribution of the change of the factor to the change in renewable energy consumption, while the contribution amount indicates the degree of contribution of the change of the factor to the change in the total renewable energy consumption).

The decomposition analysis shows that energy structure contributes the most to renewable energy consumption. The second important contributor is the low-carbon economy, and its contribution rate increases after 2012. The population effect positively affects renewable energy consumption. The population distribution effect makes a negative contribution to renewable energy consumption, but its effect on renewable energy is very small, which suggests that the population distribution effect will not affect the development of renewable energy. Energy intensity has a negative effect on renewable energy consumption, and this negative contribution gradually increases after the implementation of the Belt and Road Initiative.

### 4.3. Policy Implications

Based on the developing trends and influencing factors for renewable energy in the BRI countries, this study proposes the following policy recommendations for global renewable energy development:

(1) According to the analysis results for *β*-convergence, countries with smaller renewable energy consumption increase the level of renewable energy at a faster convergence rate. In this process, countries with higher levels should provide these other countries with relevant help, such as technology and experience. Countries with low renewable energy consumption levels should focus on urbanization and industrialization when attempting to promote the development of renewable energy. They should advocate the use of renewable energy, such as solar and wind energy in the residential sector, and formulate relevant policies for the industrial sector to increase the consumption of renewable energy. One way of achieving this goal is to provide policy subsidies and tax incentives for industries related to renewable energy development technology.
(2) Energy structure adjustment and low-carbon economic development are key factors that can be used to increase renewable energy consumption. The most important strategy for promoting renewable energy development is to control primary energy consumption and to introduce low-carbon technology, especially in the residential and industrial sectors.
(3) The renewable energy development experience in BRI countries shows that strengthening inter-regional cooperation and realizing country-to-national renewable strategy docking plays an important role in the development of renewable energy. Regional cooperation among them can utilize trade, investment and talent training, etc. Any strategic docking should strengthen exchanges between countries, implement targeted assistance and reduce tariffs on raw materials for renewable energy projects.

## 5. Conclusions 

This study investigates the developing trends and driving factors of renewable energy consumption in BRI countries. Due to data availability and validity, only 32 countries are selected as sample countries. Convergence analysis is applied to examine the developing trend of renewable energy consumption in BRI countries. Furthermore, through the expanded Kaya equation, renewable energy consumption is decomposed into energy structure effect, energy intensity effect, low-carbon economy effect, carbon emission effect, population distribution effect and population effect, and LMDI decomposition is applied to calculate the contribution of various influencing factors to renewable energy consumption. The main conclusions of this study are as follows:

(1) Renewable energy consumption in BRI countries is subject to *β*-convergence proved by the GMM estimator during 2004–2018. The results indicate that renewable energy consumption in BRI countries will converge to a relatively stable level, and countries with low renewable energy consumption will increase their renewable energy consumption with a higher convergence rate to chase the countries with high renewable energy consumption. Among the variables, per capita GDP and foreign direct investment have negative impacts on renewable energy consumption, while the urbanization and industrialization level have positive impacts on renewable energy consumption.
(2) According to the decomposition results of LMDI, renewable energy consumption is substantially increased, and energy structure is the main contributor, but the contribution rate for energy structure does not significantly change. In addition, energy intensity makes a negative contribution to the growth of renewable energy consumption, and this negative contribution rate increases after 2012. The low-carbon economy and carbon emission per capita both have positive impacts on renewable energy consumption. The Belt and Road Initiative increases the contribution made by the low-carbon economy to renewable energy consumption, but reduces the contribution made by carbon emission per capita. The contribution made by the population distribution effect to renewable energy consumption is small, while population effect makes a positive contribution to renewable energy consumption.

## Figures and Tables

**Table 1:** Control variables.

Symbol	Significance	Supporting Reviews
PGDP	Per capita GDP	The research by Sadorsky [53] indicates that per capita GDP has a positive impact on renewable energy consumption.
Ur	Urbanization	Salim and Shafiei [54] consider that urbanization makes a huge influence on renewable energy consumption in OECD countries.
FDI	Foreign Direct Investment	Doytch and Narayan [55] find that the influential extent of FDI on renewable energy consumption is distinguished between sectors.
Ind	Industrialization	Li and Lin [56] consider that industrialization has different effects on energy consumption during different economic development stages.

**Table 2:** Decomposition variables.

Variable	Definition	Data Source
*RE_i_*	Total renewable energy consumption of *i_th_* country	BP
*E_i_*	Total energy consumption of *i_th_* country	World Bank
*G_i_*	The GDP of *i_th_* country	World Bank
*C_i_*	CO_2_ emissions each year of *i_th_* country	World Bank
*P_i_*	Population of *i_th_* country	World Bank
*P*	Total population of all sample countries	World Bank

**Table 3:** *β*-convergence regression.

Variables	2004–2018			
	Model (1)	Model (2)	Model (3)	Model (4)
*L1. lnRE*	0.9417 ***	0.9221 ***	0.9297 ***	0.9199 ***
	(−0.0026)	(−0.0029)	(0.0017)	(0.0041)
*PGDP*	−0.0001 ***			
	(−0.0005)			
*Ur*		0.0112 ***		
		(0.0034)		
*FDI*			−0.0008 ***	
			(−0.0002)	
*Ind*				0.0092 ***
				(0.0018)
Constant	−0.3057 ***	−0. 4441 ***	−0.2816 ***	−0.5121 ***
	(−0.0747)	(0.2121)	(0.0137)	(−0.0394)
Wald chi2	178,668.44 ***	423,028.96 ***	181,000 ***	224,568.96 ***
Arellano-Bond test				
AR(1)	−2.2526 **	−2.2522 **	−2.2498 **	−2.2391 **
AR(2)	−1.7349	−1.7422	−1.741	−1.7494
Sargan test	31.1544	31.4869	29.82492	31.2596
Observations	385	385	385	385

Note: Standard errors are presented in the parentheses. *** indicates *p* < 0.01. ** indicates *p* < 0.05. AR(1) represents the test statistic for the first-order difference autocorrelation of residual items. AR(2) represents the test statistic for the second-order difference autocorrelation of residual items. L1. *lnRE* represents one-period lagged term of *lnRE* and is regarded as GMM-type instrument variable.

**Table 4:** Contribution rate of each factor.

Period	RS	EI	CE	PC	PP	P
2004–2005	3.69%	−17.09%	18.45%	86.29%	−5.66%	14.32%
2005–2006	20.83%	−38.30%	41.13%	68.15%	−4.79%	12.98%
2006–2007	2.53%	−61.28%	61.60%	86.64%	−5.55%	16.07%
2007–2008	73.14%	−25.26%	28.57%	18.31%	−2.65%	7.89%
2008–2009	−48.57%	−142.82%	174.76%	73.83%	−21.90%	64.71%
2009–2010	52.14%	−18.19%	26.04%	34.84%	−2.83%	8.00%
2010–2011	−104.97%	−32.01%	27.20%	188.54%	−10.38%	31.62%
2011–2012	76.65%	−17.94%	25.85%	10.89%	−2.00%	6.54%
2012–2013	71.96%	−32.33%	43.05%	10.88%	−2.91%	9.35%
2013–2014	74.25%	−34.09%	46.72%	6.43%	−3.10%	9.78%
2014–2015	75.49%	−56.08%	72.87%	−1.38%	−4.33%	13.44%
2015–2016	72.41%	−45.42%	62.66%	2.11%	−3.50%	11.75%
2016–2017	59.07%	−39.74%	57.71%	14.27%	−3.59%	12.27%
2017–2018	52.12%	−14.69%	30.18%	26.28%	−3.22%	9.33%
